# Integrated Mental Health Services for the Developmental Period (0 to 25 Years): A Critical Review of the Evidence

**DOI:** 10.3389/fpsyt.2019.00355

**Published:** 2019-06-07

**Authors:** Paolo Fusar-Poli

**Affiliations:** ^1^Early Psychosis: Interventions and Clinical-Detection (EPIC) Lab, Department of Psychosis Studies, Institute of Psychiatry, Psychology & Neuroscience, King’s College London, London, United Kingdom; ^2^OASIS Service, South London and Maudsley NHS Foundation Trust, London, United Kingdom; ^3^Department of Brain and Behavioral Science, University of Pavia, Pavia, Italy

**Keywords:** mental health, youth, development, prevention, 0 to 25, model of care, mental health services

## Abstract

**Background:** The developmental period from 0 to 25 years is a vulnerable time during which children and young people experience many psychosocial and neurobiological changes and an increased incidence of mental illness. New clinical services for children and young people aged 0 to 25 years may represent a radical transformation of mental healthcare.

**Method:** Critical, non-systematic review of the PubMed literature up to 3rd January 2019.

**Results:** *Rationale*: the youngest age group has an increased risk of developing mental disorders and 75% of mental disorders begin by the age of 24 and prodromal features may start even earlier. Most of the risk factors for mental disorders exert their role before the age of 25, profound maturational brain changes occur from mid-childhood through puberty to the mid-20s, and mental disorders that persist in adulthood have poor long-term outcomes. The optimal window of opportunity to improve the outcomes of mental disorders is the prevention or early treatment in individuals aged 0 to 25 within a clinical staging model framework. *Unmet needs*: children and young people face barriers to primary and secondary care access, delays in receiving appropriate treatments, poor engagement, cracks between child and adult mental health services, poor involvement in the design of mental health services, and lack of evidence-based treatments. *Evidence*: the most established paradigm for reforming youth mental services focuses on people aged 12–25 who experienced early stages of psychosis. Future advancements may include early stages of depression and bipolar disorders. Broader youth mental health services have been implemented worldwide, but no single example constitutes best practice. These services seem to improve access, symptomatic and functional outcomes, and satisfaction of children and young people aged 12–25. However, there are no robust controlled trials demonstrating their impact. Very limited evidence is available for integrated mental health services that focus on people aged 0–12.

**Conclusions:** Children and young people aged 12–25 need youth-friendly mental health services that are sensitive to their unique stage of clinical, neurobiological, and psychosocial development. Early intervention for psychosis services may represent the starting platform to refine the next generation of integrated youth mental health services.

## Introduction

At present, around one-fourth of the total population consist of youngsters in an age range between 10 and 24 years—the greatest proportion of this cohort in history (1, 2). When compared to their parents, the current generation faces increased complex difficulties for their well-being (3). For instance, the well-being of a great number of children and young people in human history is shaped by the exceptional worldwide forces (4). The future for this generation, and indeed for human beings, is set by population migrations, worldwide correspondences, financial challenges, and the sustainability of ecosystems (4). World Health Organization notes, “mental health disorders account for nearly half of the disease burden in the world’s adolescents and young adults” (1), in view of these changes. Mental disorders will become one of the five most familiar ailment causing dismalness, mortality, and dysfunction among youths, by 2020 (5). These mental health problems inversely sway on their academic, professional, and social activities; quality of life; and significantly impact budgetary and societal expense. As a result, the need to search for effective treatment options for mental disorders is inevitable in children and young people (6). To achieve this aim, the UK Government’s report on No Health Without Mental Health acknowledged and stressed the importance that only a lifelong approach will enable future mental health goals to be achieved (7). Correspondingly, the NHS England’s report—Future in Mind—features the urgent need (by 2020) for a holistic approach, improved access for patients, support for the forefront staff, and adoption of innovative emotional wellness programs for youth that differ from the current tier system division between Child and Adolescent Mental Health Services (CAMHS) and Adult Mental Health Service (AMHS) (8). The Five-Year Foreign View for Mental Health that set the key NHS priorities for 2020–2021 further strengthened this vision (9). These incorporated the critical requirement for equality of regard between services of physical and mental health, the necessity for children and young people to get evidence-based interventions in mental health, and the need of training staff in children and adolescence mental health interventions (9). So as to help accomplish these targets, robust evidence-based information is required not just with the involvement of local and national leadership yet additionally through a driving force on multidisciplinary teams working over all sectors. This started with a local transformation plan for NHS England fusing local partners in the NHS, public health, social services, and youth education and justice sectors to enhance mental health for children and adolescents (10). The forthcoming NHS England Long Term Plan for Mental Health is expected to rely on the mental health of children and young people between the ages of 0–25 with a view to reduce the number of young people who experience a severe mental disorder. The development of a new model of care for children and young people between 0 and 25 years will be a fundamental transformative component to improving the experience, outcomes, and continuity of care. In preparation for this objective, Healthy London Partnership (https://www.healthylondon.org/) is working close by the London Children and Young People Health Transformation Board and the Mental Health Transformation Board to consider the chances and difficulties this would go with. Against this backdrop, the current report provides an initial critical review of the literature to establish mental health services targeting the developmental period. This period includes individuals aged 0–25 years and encompassing the following phases: the perinatal period (from 22 weeks of gestation to 7 days after birth, WHO); infancy (first year of life); childhood (1–10 years); adolescence [the period of time between the onset of puberty and the cessation of physical growth, usually between 10 and 19 years (11)]; and young adulthood (particularly from adolescence on a concept of fulfilment of mental and physical capacity, usually between 19 and 25 years) (12). The main purpose of this study is to critically review the rationale, unmet needs, and evidence for developing integrated mental health services for individuals of 0–25 years of age in order to inform the ongoing developments in this field.

## Method

A critical review of the PubMed literature was undertaken up to 3rd January 2019. The articles included in this review were not selected on a systematic basis, and there is no assumption that the evidence reviewed is exhaustive. The articles were subsequently used in order to address three core subdomains that are essential to inform the development of mental health services for those belonging to the 0–25 age group: scientific rationale, unmet needs in children and young adults, and evidence for integrated mental health services for people aged 0–25.

## Scientific Rationale for Integrated Mental Health Services for People Aged 0 to 25

This section will review the core evidence that builds the rationale for establishing mental health services for people aged 0–25.

### Prevalence of Mental Disorder Across Ages

The WHO World Mental Health Survey epidemiological studies suggest that almost 50% (at least in the US) of the population will face a DSM-defined mental disorder over their life. A monotonic increase in prevalence across all mental disorders occurs between the youngest (18–29 years of age) and the higher age group (30–44 years of age), before a decline in the older age group. The exceptions to this pattern are substance use and bipolar disorders. These studies also noted that the prevalence of mental disorders is always lowest in those aged more than 60 years, accordingly suggesting that the youngest ages have an increased risk of developing mental disorders.

### Age of Onset of Mental Disorders

**Figure 1:**
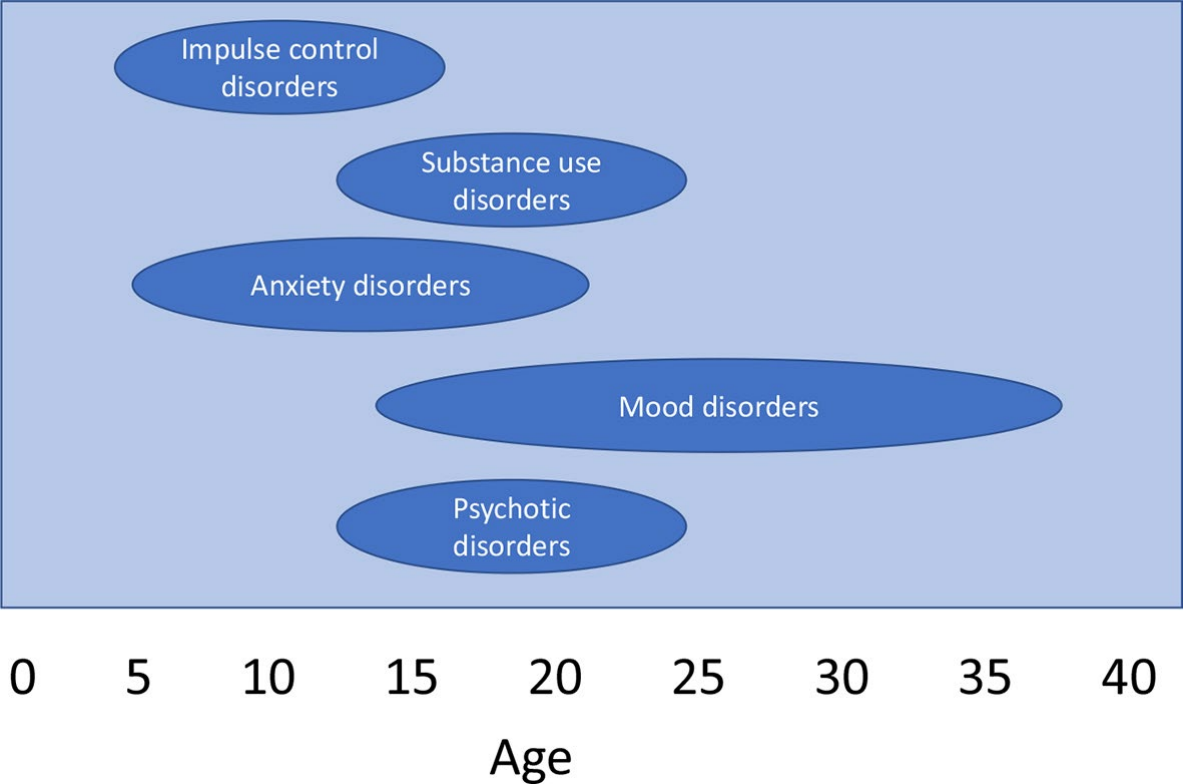
Ranges of onset age for common psychiatric disorders. Data from the National Comorbidity Survey Replication study (13), a nationally representative epidemiological survey of mental disorders. The majority of those with a mental disorder have had the beginnings of the illness in childhood or adolescence. Some anxiety disorders such as phobias and separation anxiety and impulse-control disorders begin in childhood, while other anxiety disorders such as panic, generalized anxiety and post-traumatic stress disorder, substance disorders, and mood disorders begin later, with onsets rarely before early teens. Schizophrenia typically begins in late adolescence or the early 20s [adapted from Ref. (13)].

The vast majority of mental disorders have onset in childhood, adolescence, and young adulthood (**Figure 1**). About 50% of these disorders (as shown by the 50th percentile or median in **Table 1**) start by the age of 14 (**Table 1**) and 75% start by the age of 24, with later onsets for the most part ascribed to comorbid conditions (13). Moreover, more than 80% of those with mental disorders at the age of 26 had an earlier diagnosis of any mental disorder from the age of 11; in all, 74% had a diagnosis before accomplishing 18 years old and a half before the age of 15 (12). The median onset age tends to be earlier for anxiety disorders and impulse control disorders (11 years of age) in comparison with substance use disorders (20 years of age) and mood disorders (30 years, **Table 1**) (13). Correspondingly, 80% of lifetime attention deficit hyperactivity disorders start at the age of 4–11 years, whereas most of oppositional defiant disorders and conduct disorders start in the age range of 5–15 years (14). Half of all lifetime intermittent explosive disorders begin in childhood or adolescence. Similarly, the median age of the onset of depressive disorders typically lies in the early to mid-20s, although significant proportions of depressive cases have also been known to commence during adulthood and late adulthood (15). With respect to psychotic disorders, despite being relatively rare before the age of 14 (14), their risk peaks in the age group of 15–35 and declines after the age of 35 (16). Specifically, the abovementioned studies characterize the onset of a disorder as the start of characteristics that are part and contiguous to its first expression (12). Therefore, this figure is even more dramatic when attenuated, and mild symptoms characterizing clinical risk syndromes as opposed to established mental disorders are considered (see below). In fact, the age of onset of putative prodromal symptoms is generally even sooner than that of the onset of established mental disorders (17).

**Table 1:** Ages at onset for five categories of mental health disorder [adapted from Ref. (12)].

		Age at which % of projected lifetime risk attained		
	Projected lifetime risk%	25%	50% (median)	75%
Anxiety disorders	32	6	11	21
Mood disorders	28	18	30	43
Impulse control disorder	25	7	11	15
Substance use disorders	16	18	20	27
Any disorder	51	7	14	24

### Developmental Pathophysiology of Mental Disorders

**Figure 2:**
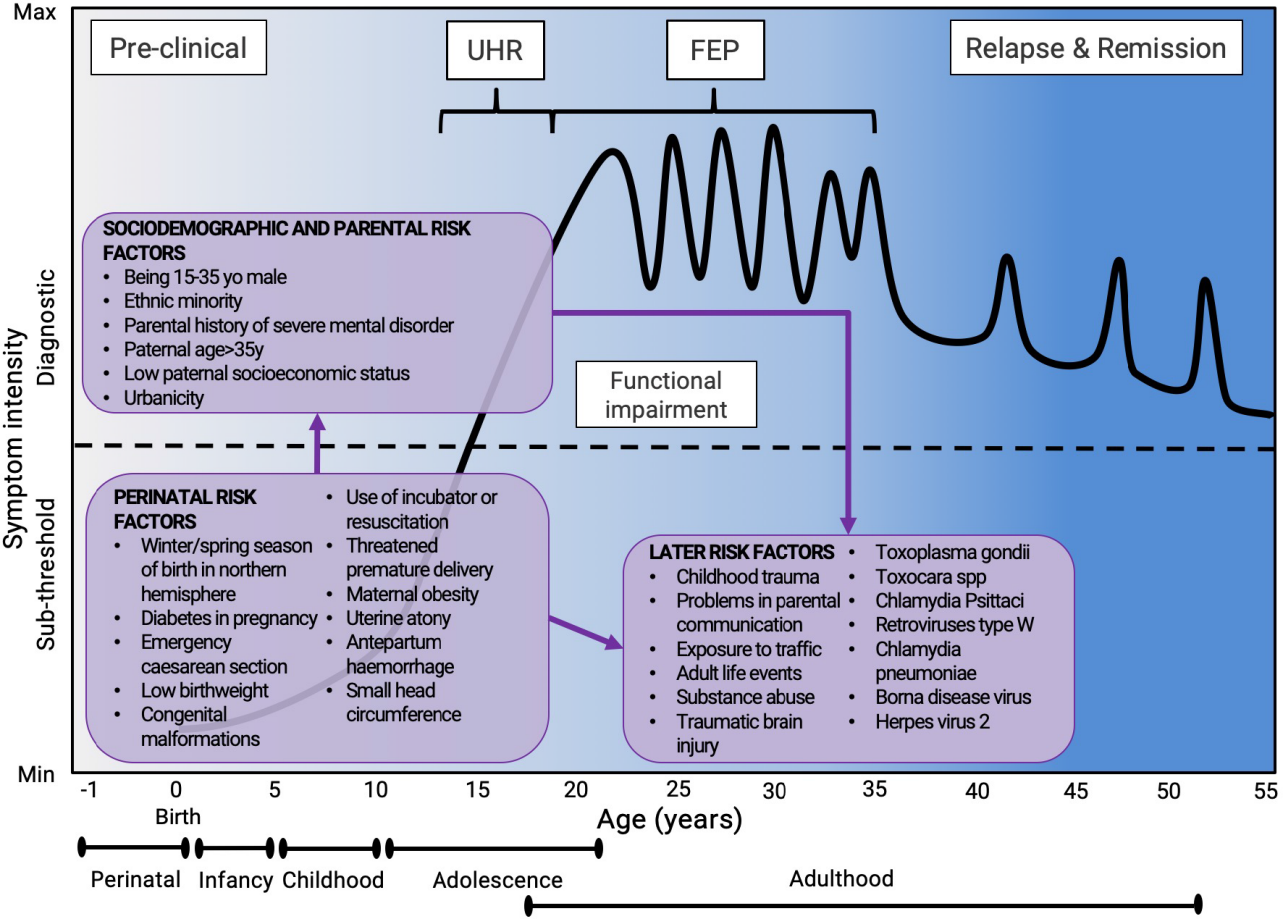
Putative model of the onset and progression of psychosis in relation to non-purely genetic risk factors and developmental processes affected by the disorder. Sociodemographic and parental risk factors and perinatal risk factors have been implicated during the preclinical phase, usually observed from birth to infancy, childhood, and early adolescence. Additional later factors occurring during later adolescence and early adulthood can trigger the onset of attenuated psychotic symptoms, functional impairment, and help-seeking behavior, which constitute the CHR-P stage. The diagnosis of psychosis, which operationally corresponds to the first episode of psychosis, is usually made during the adolescence or early adulthood, with a peak from 15 to 35 years. Once diagnosed, psychosis usually follows a fluctuating course punctuated by acute exacerbation of psychotic crises superimposed upon a background of poorly controlled negative, neurocognitive, and social cognitive symptoms. The pink boxes represent the risk factors for psychosis (16). FEP, First Episode Psychosis; CHR-P, Clinical High Risk for Psychosis.

The model to have received the strongest empirical support for elucidating the pathophysiology of mental disorders implicates direct genetic and environmental effects alongside their interactions. For instance, as delineated in **Figure 2**, schizophrenia diagnosis corresponds to the first episode of psychosis. The diagnosis is usually made in young adults but can (although rarely) also happen in childhood, adolescence, or later in life. Generally, diagnosis of a first episode of psychosis is preceded by a clinical high-risk stage (17, 18) in which attenuated psychotic symptoms (19), functional impairment (20), and help-seeking behaviors (21), are evident. Schizophrenia, following the first episode, pursues a fluctuating course marked by the intensification of psychotic crises that are surrounded by negative psychotic symptoms, neurocognitive deficits, and alterations in social cognition. After their first episode, about 10–15% of patients recover, with a comparable extent showing an increasingly severe and unremitting form of the disorder. Beyond genetic inheritance, numerous environmental risk factors for the onset of psychosis have been implicated during the perinatal (first-wave) and adolescence (second-wave) period (16, 22). As portrayed in **Figure 2**, the majority of these risk factors exert their role before the age of 25 years. Genetic and environmental factor impacts the epigenetic misprogramming of neurodevelopment (see below), amid this period. Importantly, some risk factors for psychosis, such as the perinatal risk factors, can impact the course of the disorder during the very early phases of the development. This lays the rationale for intervening at the time of birth (age 0) to impact the course of psychotic disorders. Finally, the model represented in **Figure 2** can be adapted to other mental disorders, some of which (e.g., autism spectrum disorders or attention deficit hyperactivity disorder) are intrinsically neurodevelopmental.

### Neurobiological Changes During the Developmental Period

Neurobiological research shows that the human brain reflects this tides of risk factors and incident mental disorders during the developmental period of children and young people (12). Mental disorder pathophysiology is progressively understood to originate from abnormalities of maturational changes that regularly happen in the developing brain from the time of birth. Notably, these maturational changes are known to affect brain structure, brain activity, pruning and myelination processes, neural connectivity, and neurochemistry (23). Development of the neonatal brain from its ectodermal phase is a dramatic accomplishment of nature. Complex and predicated on different mechanisms, this period is particularly susceptible to neurodevelopmental disorders and learning delays. The core processes that may be disrupted include the development of brain connectivity and programmed cell death, followed by fundamental cabling through myelination amid the first year (12). It takes as long as three decades to grow a mature human brain; much further development takes place during this period (12). In the meantime, there is a further phase of significant neurobiological and behavioral changes from mid-childhood through pubescence to mid-20s, especially in the connectivity balance between the brain areas (12). These maturational changes are normally useful, optimizing the brain for the challenges ahead but may at the same time increase the vulnerability to emerging mental disorders (23). Indeed, the risk of adult mental health disorders is the highest during this period. In addition, this maturation gap may also present a vulnerability window, which does not yet fully coordinate different brain mechanisms and systems (12).

**Figure 3:**
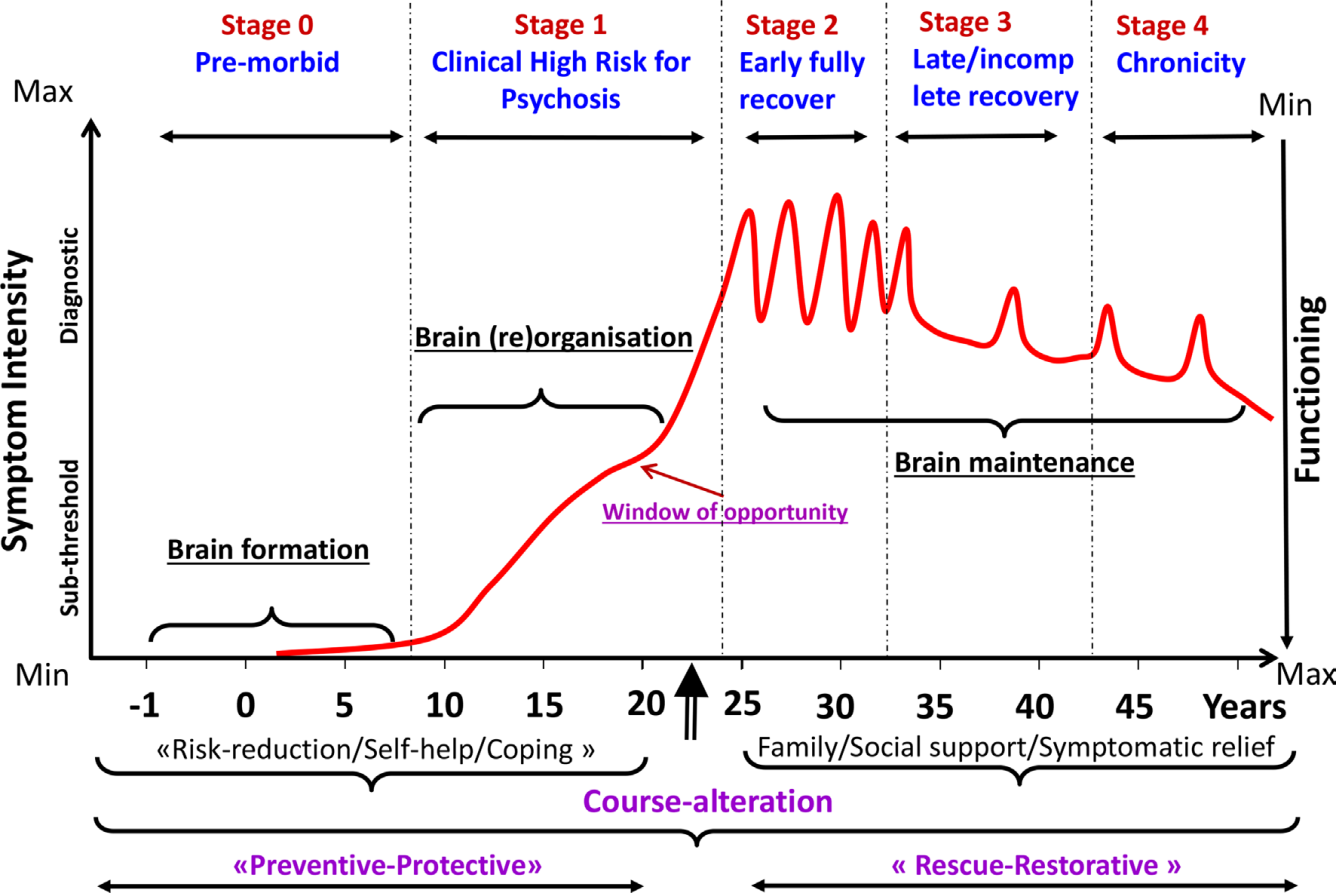
Onset and progression of psychosis in relation to the developmental processes affected by the disorder [adapted from Ref. (25)]. During the premorbid and clinical high risk for psychosis neurodevelopmental phases, risk reduction strategies can exert the highest impact for course alteration. During the early fully recover/late incomplete recovery and chronicity phases, rescue and restorative strategies can have the highest impact on course alteration.

The relationship between maturational changes and emerging psychopathology can be conceptualized as “moving parts get broken” (23), but this relationship is not a unitary concept; instead, it is specific to each type of mental disorder. For example, the course to and the progression of psychosis illustrated in **Figure 3** match the effects of risk factors for psychosis depicted in **Figure 2** and can be identified with three key stages in the “life” of the brain. In spite of being delineated consecutively, these three stages are interlinked, and there is no outright division. Also, each phase in psychosis is anomalous, with brain formation disruption and reorganization phases involved in causal pathophysiology. These two stages as well as brain maintenance encompass a range of mechanisms, which might be potentially targeted by preventive interventions. Similar neurodevelopmental models have been postulated for other mental disorders, including depression (24).

Overall, neurobiological research clearly indicates that the brain’s developmental period represents the most important window of opportunities to impact the development of the brain and, as such, improve the outcomes of mental disorders. From the viewpoint of brain development, mental health services obviously require re-engineering to give a properly consistent and developmentally sensitive way to deal with children and young people during the two-decade venture from adolescence to adulthood (12).

### The Course of Mental Disorders

It does not seem surprising that most adult mental disorders have a genesis in childhood, adolescence, or young adulthood, as developmental physiology and brain change occur during this period. We may then wonder what the longitudinal outcomes from these disorders are. Although certain incident disorders will resolve, it is obvious that many do persist, bringing lifelong disability and forcing substantial cost burden on society and the individual (12). The majority of mental health disorders associated with the personal burden that manifest at the age of 26 should be considered as extensions of adolescent disorders (16). Besides, in spite of the fact that the onset of the disorder at a very young age is commonly connected with a good response to treatment (12), these disorders accrue additional comorbidity once they persist into adulthood, especially if left untreated. Thus, their response to treatment becomes poorer in the later stages. For example, once psychotic disorders develop and become chronic, there are only limited treatment possibilities to improve their outcomes (26) (refer to the clinical staging model below). By and large, these discoveries recommend that it is fundamental to coordinate endeavors on early recognition and treatment targeting the developmental period that represents the most important window of opportunity to reduce the burdens and poor consequences of mental disorders. As illustrated in **Figure 3**, the most compelling “window of opportunity” to improve the outcomes of psychotic disorders is around the first episode of the disorder, to hinder it onset or stop early progression (25). According to these findings, the eradication of mental disorders presenting during the developmental period, through interventions aimed at prevention or early treatment in youths, would have a profound impact on reducing subsequent morbidity and chronicity (13).

### Clinical Staging of Mental Disorders

Overall, the robust findings from modern epidemiology (prevalence and age of onset of mental disorders) and their compliance with the emerging pathophysiology, neurobiology, and course of the developmental period should represent a strong rationale for preventive and early intervention. Notably, the clinical staging model of mental disorders accommodates all these features to pragmatically facilitate preventive treatments and early interventions for youths. This clinical staging model was first proposed in psychiatry 25 years ago (in 1993) (27), before being subsequently adapted for psychotic disorders (28) (in 2006) to overcome the limitations of the standard ICD or DSM diagnostic systems. Clinical staging was put forward as a “simply more refined form of diagnosis” with two core fundamental assumptions: individuals experiencing an early phase of a disorder show a superior response to treatment and better prognosis, and the treatments offered during the early stages are more benign and effective (28). The main advantages of the clinical staging model are to accommodate the previously mentioned developmental findings, to facilitate preventive strategies to impede the progression to more advanced stages, or to facilitate the regression to an earlier stage and thus bolster better clinicopathological research (28).

**Figure 4:**
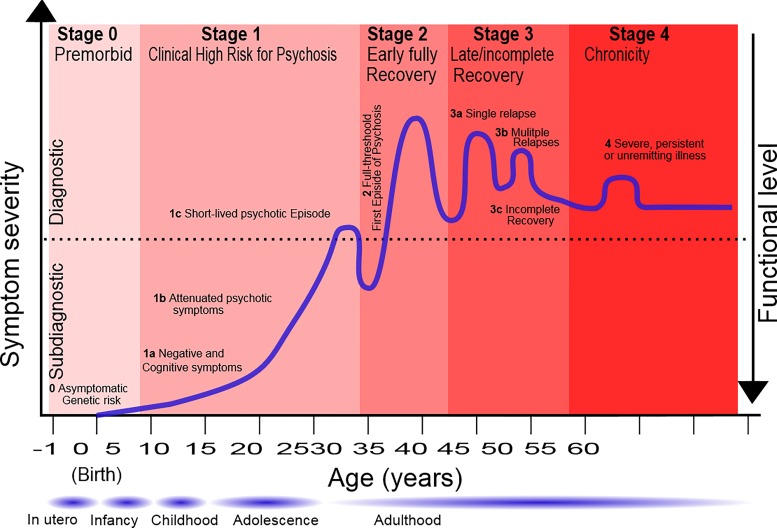
Clinical staging of psychotic disorders. Unpublished figure courtesy of Paolo Fusar-Poli. The age bounds indicated are only descriptive. Stage 0 (premorbid) is followed by the clinical high-risk stage 1 for psychosis and then by stage 2 (early fully recover). Stage 3 describes a late/incomplete recovery and stage 4 is the chronic phase of psychotic disorders. Substages 1a–c and 3a–c are also indicated in the figure.

For example, after about two decades of research into the clinical staging model in psychosis, its definition and impact have recently been reviewed (26). As summarized in **Figure 4**, stage 0 may allow primary selective prevention in asymptomatic subgroups. Meanwhile, stage 1 would allow primary selected prevention in patients who have an increased likelihood of developing psychosis (i.e., those with negative and cognitive deficits: stage 1a; with attenuated psychotic symptoms: stage 1b; or with short-lived psychotic episodes: stage 1c) (26). At the time of the first episode of psychosis (stage 2), early intervention and secondary prevention strategies can minimize the duration of untreated psychosis, improve treatment response and adherence, reduce illicit substance abuse, and prevent relapses (26). Meanwhile, at the time of an incomplete recovery (stage 3, which includes single relapses: stage 3a; multiple relapses: stage 3b; and incomplete recovery: stage 3c), early intervention and tertiary prevention strategies can improve treatment resistance well-being and social skills, reduce the burden on the family, improve treatment outcomes of comorbid substance use, and prevent multiple relapses and disease progression (26). During the chronicity stage, i.e., stage 4, the key treatment focuses on maintenance treatment (26). Similar clinical staging models are also emerging for other mental disorders, such as bipolar disorders (29) or depressive disorders (30). Since clinical staging models for psychosis, bipolar disorders, or depressive disorders share some similarities, some authors have proposed an overall “transdiagnostic” clinical staging model that cuts across different diagnostic spectra (31, 32). However, the internal coherence of transdiagnostic approaches in psychiatry and their pragmatic advantages as compared to diagnostic-specific approaches to date have remained unclear [for a recent systematic review on transdiagnostic approaches in psychiatry, see Fusar-Poli et al. (33)].

In summation, the rationale for establishing mental health services for people aged 0–25 is premised on the following compelling pieces of evidence:

- The youngest age group has an increased risk of developing mental disorders;
- 75% of mental disorders begin by the age of 24;
- Putative prodromal features that precede mental disorders start even earlier;
- Most of the risk factors for mental disorders exert their role before the age of 25;
- Some risk factors exert their role during the perinatal period (age 0);
- Profound maturational brain changes occur from mid-childhood following puberty and finally mid-20s;
- Mental disorders can persist in adulthood with poor long-term outcomes;
- The most optimal window of opportunity to improve the outcomes of mental disorders is during the developmental period;
- Prevention or early treatment in individuals aged 0–25 may eradicate or at least improve the outcome of mental disorders during adulthood;
- The clinical staging model leverages the aforementioned points to allow early detection and intervention for young people with emerging mental disorders.

## Unmet Mental Health Needs in Children and Adolescents

This section will review to what extent current mental health services meet the scientific rationale detailed above in order to improve the mental health of individuals aged 0–25.

### Barriers to Access

While 75% of psychiatric disorders, in general, develop before the age of 25, and the biggest burden of such disorders is on young people, the paradox is that they have the worst level of mental healthcare access throughout their entire lifespan (34). The gap between the prevalence of mental disorders in children and young people and treatment rates is therefore obvious, with only 25–35% of children and young people affected accessing treatment (6). Indeed, youngsters find it hard to access mental health services (8). The existing tier system for CAMHS is rigid and calls for children and young people to fit into the services, as opposed to services that respond to their needs (35). On the other hand, innovative healthcare options are needed in an increasingly modernized and digitalized world in order to promote and maintain engagement with children and young people, by involving them in service users groups, by transmitting practice news in social media, and by enlarging the utilization of digital healthcare innovation as a way to better connect with young people. A recent review demonstrated that the youngsters have uninformed and stigmatizing convictions about mental healthcare, mental health professionals, and access to care (36), which substantially curtail their abilities to look for help where they most need of it.

### Delays to Initial Treatment

Analysis of service contact data from epidemiological studies investigations passes on a troubling story of disappointment, postponement, and lost opportunities (37, 38). The large majority of young individuals with lifelong mental disorders eventually reached mental health services, though more commonly for mood disorders than for anxiety, impulse controls, or substance use disorders (12). Treatment delay among those who in the long run made contact with mental healthcare ranged from 6 to 8 years for mood disorders (39). In this regard, a recent meta-analysis has identified a delay of 6 years between the onset of bipolar disorder and the initiation of a treatment (39). Delay to the initiation of treatment ranges from 9 to 23 years for anxiety disorders (12). Failure to establish initial contact with mental healthcare and delay in receiving treatment among those who finally made contact with services were associated either with early onset age or with sociodemographic characteristics such as being male, poorly educated, or black/minority ethnicity (12).

### Poor Engagement With Mental Health Services

When youngsters gain access to mental health services, they experience consistent delays in receiving appropriate care. The situation is exacerbated by the fact that the retention rate for those who are eventually offered some treatment remains poor. According to a meta-analysis, a vast extent (up to 75%) of the treatments in children and young people leads to premature termination (dropout) (40). Both ethnic minority status and socioeconomic status have been established as risk factors for dropping out (41) and males are at particularly high risk of disengagement (42).

### Barriers to Primary Care

General practitioners in primary care play a vital “gatekeeper” role to specialist mental healthcare for children and young people (6, 43). Commonly, the average British kid consults their general practitioner at least once a year (6). Children and adolescents presenting to their general practitioners are twice as likely to develop a mental health problem (35). A survey made in 2016 across 302 general practitioners reported that 78% of general practitioners are seeing more children and adolescents with mental illness, and 61% are seeing more self-harming young people than they had 5 years ago (35). However, primary care professionals experience difficulties in both the recognition and management of mental health problems (6). For example, children and young people manifest symptoms of mental disorders differently from adults, may frequently present with physical symptoms, or may not be as forthcoming with their issues (6). Waiting times also tend to be longer, and 89% of general practitioners express concerns over exposing children and young people to risk while waiting for inputs from a specialist (35). These issues are additionally exacerbated by the fact that consultation time in primary care is ordinarily short. In the UK, for instance, patients talk to primary care practitioners about their mental health problems for just 9 min on average per consultation (6, 44). Primary care practitioners likewise face additional difficulties after having identified the presence of a psychological well-being issue. In fact, only a minority of children and young people are eventually able to access specialist mental health services (6, 45), typically those belonging to a majority ethnicity, with a higher parental perceived burden or greater symptom severity (6). Moreover, the individuals who do get referred onwards are frequently subject to significant delays in receiving specialist care, as observed above. A recent systematic review concluded that the paucity of specialist service providers for youths was the most highly endorsed barrier by primary care practitioners (6).

### Falling Through the Cracks

Current mental health services have developed without the new clinical staging model knowledge that psychopathology and brain maturation sees no transition among adolescence and early adulthood (12). Therefore, access to mental health services has been driven by a historical paediatric–adult bifurcation in which CAMHS services are usually cut at the age of 18 (the transitional period) (34), when young people are the most liable to mental disorders and are at the greatest risk of decreased use of healthcare services (2). Indeed, only a minority of young people below the age of 18 can access these limited specialized services (34). Simultaneously, AMHS services are unable to take into account the needs of young people with emerging mental disorders (34). These services are developmentally inappropriate for young individuals since they center around older patients with more severe and persistent mental disorders and thus overlook the presence of less serious young adults (34). Young people with emerging mental illness or at-risk syndromes (discussed later) typically present with blurred and unspecific symptoms that do not fulfill the adult-type diagnostic criteria, which additionally limit their eligibility to receive AMHS care (46). Furthermore, an absence of clear linkage or pathway is often noted between CAMHS and AMHS. Inconsistencies in service provision and practice standards for continuity of care during the transitional period from CAMHS to AMHS also lead many youths to fall through cracks (47). The assumption that the transition from CAMHS to AMHS is easily possible for adolescents and their families—considering all of its concomitant complexities without embedded supports and coordination of care pathways—is misplaced (2). Research-based evidence from Australia, Canada, the UK, and the United States have confirmed that it is highly difficult to provide coordinated/integrated youth services during the transitional period (47). The transition is frequently portrayed by complexity because it associates with the peak of risk for the onset of mental disorders that requires a variety of community and vocational packages of care to meet the multifaceted needs of youths (47). For many governments and institutions all over the world, continuity of care for youths transitioning between CAMHS and AMHS who require mental healthcare has been identified as a top priority. These transitional health services are innately complex, and their organization and function can vary according to geographic, administration, types of delivery, financing, and service type. Within this complexity, an important element is the subjective experience of youths during the transitional period. Young people experience a deep emotional culture shift when transitioning from CAMHS to AMHS. Similarly, their carers may feel invisible and often in distress, with several of them reporting mental health problems arising from their experience of caring (9). At the same time, young people and their carers express important subjective views to direct the development and design of youth-friendly mental health services. Therefore, it seems imperative to incorporate the perspectives of young individuals into transitional service improvement (48). A final problem is the current division of training, which leads to different and often contrasting diagnostic and treatment approaches for CAMHS vs. AMHS clinicians, which may additionally enhance the cultural and pragmatic divide among the specialities and promote a silo approach to care (49). Collectively, the above system weaknesses create a barrier to children and young people receiving mental healthcare, resulting in missed opportunities for timely intervention.

To summarize, children and young people are currently encountering substantial unmet needs due to the following reasons:

- Barriers to access;
- Delays in receiving appropriate treatments;
- Poor engagement with mental health services;
- Up to 75% treatments leading to premature termination;
- Limitations to the gatekeeper role of primary care;
- Cracks between CAMHS and AMHS;
- Poor involvement in the design of mental health services;
- Lack of incorporation of scientific evidence into clinical care (clinical staging and early intervention during the developmental period).

## Evidence for Mental Health Services for People Aged 0–25

This section will review different models of care and configurations of mental health services along with their impact on the unmet needs of those aged 0–25. More specifically, we pragmatically define a “model of care” as an integrated youth-specific, stigma-free early intervention service that is developmentally appropriate (34). This endeavor aims to improve access to services and patient outcomes over the years most at risk for emerging mental illness, thereby obviating the need for a transition from CAMHS to AMHS services during this critical phase (34). This ideally implies the establishment of a youth mental health healthcare model that encompasses and interacts it, but is particular from healthcare systems for children and young people.

## High-Order Principles Governing the Development of Youth-Friendly Health Services

High-order principles have been published for the development of youth-friendly health services. These include the following: addressing inequities (including sex disparities) facilitating the regard, insurance, and satisfaction of human rights, as stipulated in internationally agreed human rights agreements such as the Millennium Development Goals and the UN Convention on the Rights of the Child (which likewise underpins the more explicit attributes of youth-friendly services, for example, youth participation and confidentiality). The characteristics of youth-friendly healthcare services have been fully described in the context of the WHO’s guiding program development (**Box 1**).

Box 1 **WHO framework for development of youth-friendly health services [from Ref. (3)].**

**An equitable point of delivery is one in which:**

- Policies and procedures are in place that do not restrict the provision of health services on any terms and that address issues that might hinder the equitable provision and experience of care
- Healthcare providers and support staff treat all their patients with equal care and respect, regardless of status

**An accessible point of delivery is one in which:**

- Policies and procedures are in place that ensure health services are either free or affordable to all young people
- Point of delivery has convenient working hours and convenient location
- Young people are well informed about the range of health services available and how to obtain them
- Community members understand the benefits that young people will gain by obtaining health services, and support their provision
- Outreach workers, selected community members and young people themselves are involved in reaching out with health services to young people in the community

**An acceptable point of delivery is one in which:**

- Policies and procedures are in place that guarantee client confidentiality
- Healthcare providers
  - provide adequate information and support to enable each young person to make free and informed choices that are relevant to his or her individual needs
  - are motivated to work with young people
  - are non-judgmental, considerate, and easy to relate to
  - are able to devote adequate time to their patients
  - act in the best interests of their patients
- Support staff are motivated to work with young people and are non-judgmental, considerate, and easy to relate to the point of delivery:
  - ensures privacy (including discrete entrance)
  - ensures consultations occur in a short waiting time, with or without an appointment, and (where necessary) swift referral
  - lacks stigma
  - has an appealing and clean environment
  - has an environment that ensures physical safety
  - provides information with a variety of methods
- Young people are actively involved in the assessment and provision of health services

**The appropriateness of health services for young people is best achieved if:**

- The health services needed to fulfil the needs of all young people are provided either at the point of delivery or through referral linkages
- Healthcare providers deal adequately with presenting issue yet strive to go beyond it, to address other issues that affect health and development of adolescent patients

**The effectiveness of health services for young people is best achieved if:**

- Healthcare providers have required competencies
- Health service provision is guided by technically sound protocols and guidelines
- Points of service delivery have necessary equipment, supplies, and basic services to deliver health services

Six groups of youth-friendly health services can be delineated. The first type is the health service that is specialized in children and adolescent care in a hospital setting. The second type is a similar specialized service but located in the community. The third type is school- or college-based and stakeholders connected with schools or universities. The fourth type is a community-based center that not only provides health services but also provides other services such as educational support. The fifth type of health services includes pharmacies and shops that sell health products but do not provide health services. The sixth type is based on outreach information on the provision of services. The point of contact for this type of service is in spots where children and young people assemble—work or in schools (3).

A large portion of these principles and configurations have been used and adapted so as to guide the advancement of youth-friendly mental health services.

### Perinatal Mental Health Services

Perinatal mental health services have evolved over time. Initially, they were bound to a close interest in severe forms of postpartum psychosis (50), to encompass, during the most recent years, non-psychotic mental disorders (51), the broader mental health of women, and the neurodevelopmental course of the fetus and infant (52). For example, the identification and management of women affected with postnatal depression became an important public health target, with screening programs being developed in several countries (53). Usually, perinatal mental health services offer care from the time of conception until the end of the first postpartum year (54). The origin of perinatal psychiatry, as a medical speciality (1980), can be associated with the development of the first psychiatric units that allowed the joint admission of mothers and babies (mother and baby units) (54). These units have clear benefits because they maintain mothers and their babies in near proximity, thus alleviating the family burden and ameliorating maternal competence. These benefits, in turn, would support the development of the newborns (54). An associated relevant clinical issue has been the safety of prescribing antipsychotics, mood stabilizers (55), and other psychotropic molecules during pregnancy and for nursing mothers. Recognizing the advancements in perinatal psychiatry, some countries such as the UK and Switzerland have developed perinatal mental disorders, in order to improve mental health services for perinatal women and ensure adequate treatment (56). However, to date, perinatal mental health services have not been fully integrated into preventive approaches for the developmental period.

### Primary Indicated Prevention of Psychosis in Those at Clinical High Risk

The building blocks for reforming youth mental services began with the management of young people who experienced early stages of psychosis (26). This model of care has been unequivocally successful in the UK as well as worldwide. It entails the primary indicated prevention of psychotic disorders in people at clinical high risk for psychosis—such as those meeting the At Risk Mental State criteria (57)—and early treatment of individuals presenting with a first episode of psychosis (26). Individuals who are at clinical high risk for psychosis are detected and evaluated with established psychometric tools that have been validated in the 8–40 age group, although the most frequent age range for this population, at least in the UK, is 14 to 35 (17). Subjects at clinical high risk for psychosis display subtle features and overall functional impairment (20). These problems impel them to seek help at specialized clinics (58). One of the largest and oldest of these clinics is the Outreach and Support in South-London (OASIS) clinic, at the Maudsley NHS Foundation Trust (58). **Box 2** illustrates the clinical care provided at the OASIS, which crucially involves the development of extensive collaborations between AMHS and CAMHS. Individuals at clinical high risk for psychosis are 20% likely to develop emerging psychotic disorders (but not other non-psychotic disorders (59, 60)) over a relatively short period of 2 years (61). While primary indicated prevention in people at high clinical risk can alter the course of psychosis and reduce the duration of untreated psychosis, secondary prevention in those people can ameliorate the severity of the first psychosis episode (26, 62). Furthermore, tertiary prevention of relapses or other adverse clinical outcomes/behaviors in patients experiencing a first episode of psychosis can improve their long-term outcomes (63–65).

Box 2 **Case study from the Outreach and Support in South-London (OASIS) service, which takes care of young individuals aged 14 to 35 who may be at risk of developing psychotic disorders. The clinical case is taken from Ref. (46).**

**Presentation**

A 16-year-old boy was referred from the general practitioner to the local CAMHS owing to a drop in functioning and social withdrawal during the previous 6 months. The CAMHS then referred the patient to the OASIS, which managed to assess him within 5 working days. The patient began college 6 months prior but had found the workload difficult and failed his examinations. He had no family history of mental disorders, denied any current or past use of drugs, and reported no significant medical history. At the time of the OASIS assessment, he was well kempt, was quiet during his interview, and provided short answers. He reported that he no longer enjoyed his former interests and could not relate to people at college or to friends, but there were no clear signs of depressive disorders. No formal thought disorders were elicited. He was 80% convinced that random people looked and talked about him when he was out in public, but was able to question it. He stated that these people were probably commenting on the way he looked, but he did not believe these individuals meant him harm. He never acted on these thoughts. He also reported a vague feeling of perplexity and derealization. These experiences began when he started college and continued to occur every day for up to an hour at a time, causing significant distress. The Structured Clinical Interview for *DSM* did not reveal any mental disorder and, as such he would not be eligible to receive the care of local mental health services.

**Diagnostic and prognostic formulation**

Diagnostic designation: clinical high risk for psychosis (CHR), attenuated psychotic symptoms subgroup, determined using the Comprehensive Assessment of At-risk Mental States (CAARMS). Prognosis: the increased risk of developing psychosis is 26% at 3 years (95% CI, 23%–30%).

**Clinical care**

First, the OASIS shared with the CAMHS the result of the prognostic test. Over the past two decades, the OASIS has developed specific co-working agreements with the local CAMHS to optimize the care of children and young adults during their transitional period. These co-working agreements are particularly useful in avoiding crisis-driven connection between CAMHS and AMHS at points of heightened illness severity such as the transition from a CHR state to full-blown psychosis. At the same time, the result of the prognostic assessment was shared with the patient in the context of psychoeducational support offered by the OASIS. Informing patients about their risks is an essential component of preventive approaches in all branches of medicine. For example, individuals who meet CHR criteria accumulate several risk factors for psychosis, some of which may be potentially modifiable. The second clinical action of the OASIS was to recommend close clinical monitoring for adverse clinical outcomes during the ensuing 3 years, because this is the peak of risk. Finally, the patient was offered specific preventive interventions (indicated primary prevention) that were based on psychological therapies (cognitive behavioral therapy) and that are routinely provided by the OASIS, in line with the NICE recommendations. These treatments aim to improve the presenting symptoms and disability and to stop the progression to psychosis.

**Outcome**

When the patient turned 18, the OASIS took full clinical responsibility of him continuing the clinical monitoring and preventive interventions. At 3-year follow-up, the patient had not developed psychosis. He fully recovered from his initial problems, completed his college examinations, and was able to enjoy his social life. He expressed high satisfaction with the quality of care received by the OASIS.

The impact of primary indicated prevention in patients between 14 and 35 of age who are at clinical high risk for psychosis has been so relevant that NHS England implemented a new Access and Waiting Times-Standard for Early Intervention in psychosis (AWT EI Standard) in April 2016 to extend the prevention of psychosis across England. The Standard mandates an evidence-based nationwide detection and rapid treatment of patients at clinical high risk for psychosis aged 14–35. Therefore, the NHS requires all suspected patients presenting to early intervention services in England to be assessed and interviewed for a potential state of clinical high risk for psychosis (66). Early intervention services have grown to about 150 serving about 1000 people per month in England, and they are far more developed as compared to the rest of Europe. Early intervention services for people experiencing a first episode of the disorder are universal in England and are also available in other parts of the UK. While there are some stand-alone clinical high-risk services in the major cities, assessment and treatment of clinical high risk patients are confined to the remit of first episode services in the absence of a dedicated clinical high-risk team. The major cities in England will witness clinical high risk and first episode of psychosis services. Furthermore, several academic sites with diverse and complementary skills are conducting extensive research on clinical high-risk patients in the UK. For example, a new National Institute of Health Research-Mental Health Translational Research Centre (NIHR-MH TRC) has recently been established to facilitate clinical research in the UK. The NIHR-MH TRC includes a specific workstream on early psychosis, which will facilitate the early detection and intervention in individuals aged 15–35 who may be at risk of psychosis or experiencing a first episode of psychosis. Therefore, the UK has unparalleled central resources for early detection and treatment of individuals who are experiencing emerging serious mental disorders throughout the developmental period. This could serve as an ideal platform to further refine the development of youth mental health services for those aged 0 to 25. For example, the UK early intervention for psychosis platform could be broadened to incorporate early detection and intervention approaches for depression in young people aged 12–25 years old (67). In fact, when early interventions for depression are restricted exclusively to children and adolescents, they will miss much of the early symptoms of depression because the age of onset of this disorder—as reviewed above—overlaps with young adulthood (67). The upper limits of age eligibility, therefore, curtail continuous care. In addition to lessening the effect of depression, the provision of indicated primary prevention for depression is also known to ameliorate access to care (67). The UK early intervention for psychosis platform could additionally include early intervention in bipolar disorder, which is gaining momentum (68). New psychometric instruments have been developed in order to identify young people aged 14–35 who may be at risk of developing bipolar disorders (69) and preventive treatments are under development.

### One-Stop Early Intervention Services: Headspace

**Figure 5:**
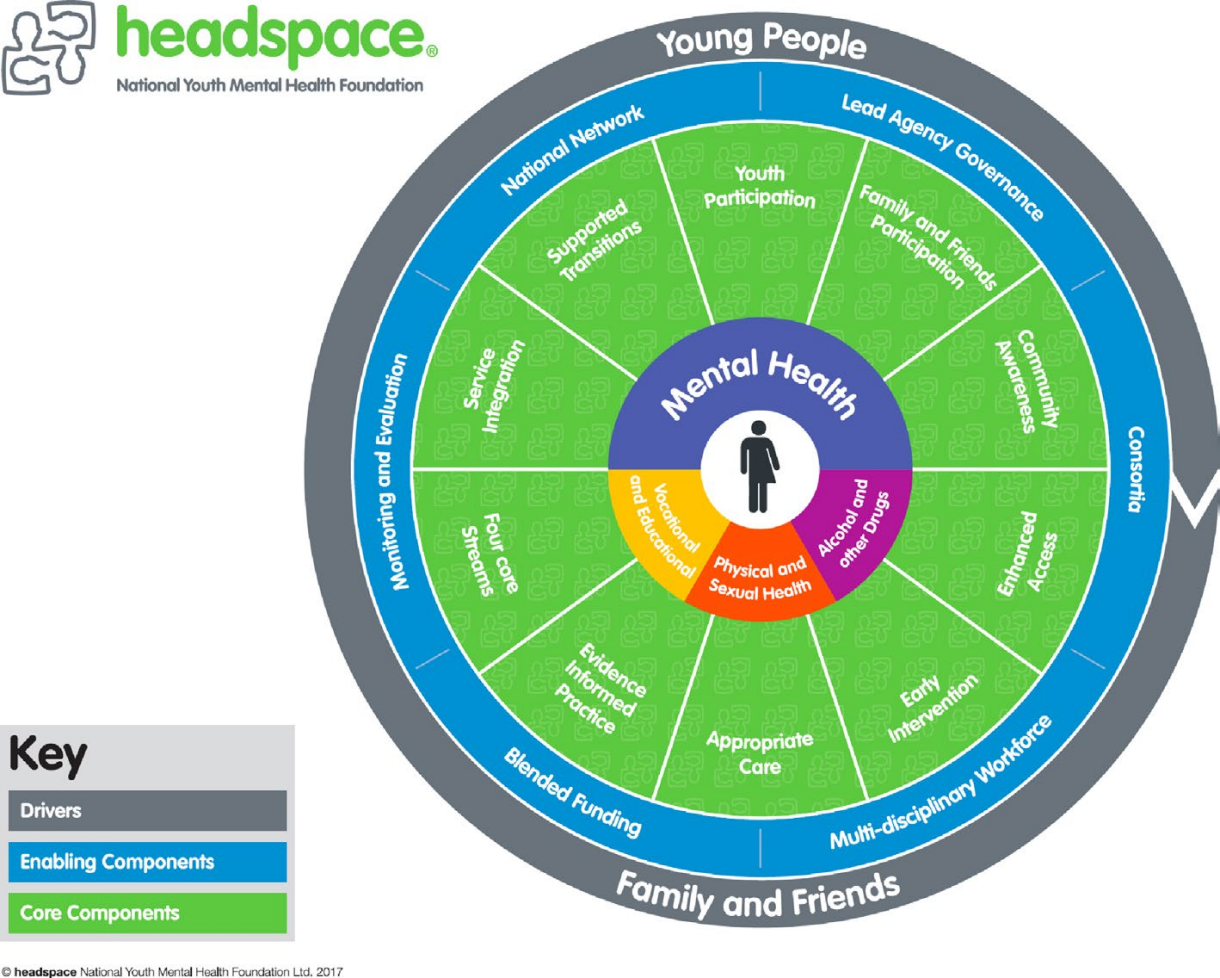
The needs of young people and their families are the main drivers of the Headspace integrated mental health model for children and young adults. Headspace has 10 service components (youth participation, family and friends participation, community awareness, enhanced access, early intervention, appropriate care, evidence informed practice, four core streams, service integration, and supported transitions) and six enabling components (national network, lead agency governance, consortia, multidisciplinary workforce, blended funding, and monitoring and evaluation). Through implementation of these core components, Headspace aims to provide easy access to one-stop, youth-friendly mental health, physical and sexual health, alcohol and other drug, and vocational services for young people across Australia [from Ref. (71)].

Some integrated models of care have already leveraged the early psychosis field to broaden their horizons and target the wide mental health of children and young adults. The early intervention model of psychosis was broadened to include further diagnoses (e.g., mood disorders, eating, substance use, and personality disorders), following a campaign led by leaders in the mental health field in 2006. This was accomplished through the formation of Headspace in Australia (https://headspace.org.au) (34). Headspace is a governmental program providing stigma-free early intervention services configured in a “one-stop shop” location for people 12–25 years old with emerging mental disorders (34). The Headspace model of care is multidisciplinary, integrated, and delivered in a single setting that constitutes a soft entry point to mental healthcare. The Headspace model is centered on the needs of young people along with their families (70). Building up the Headspace program required the formation of a new mental health service to envelop four key domains: mental health, physical health, drug and alcohol interventions, and educational support (34). As mentioned above, young people’s engagement is a central part of this healthcare model and helps to create a non-stigmatizing environment. This is achieved by ensuring the provision of Headspace services in an accessible setting, non-judgemental and young people-friendly (34). **Figure 5** summarizes the essential clinical components of Headspace. The success of Headspace is evidenced by the fact that it has grown from 10 centers to over 110 in 2018 (34). These centers are accessed by about 100,000 youngsters every year, and an extra 30,000 youngsters are accessing its online service platform through eheadspace (34). In the recent assessment, the authors have reported that a range of young people with high levels of psychological distress was able to access Headspace (34). Importantly, these young individuals included vulnerable groups (34). Headspace was likewise observed to be effective in diminishing suicidal ideation and self-harm, as well as in reducing the quantity of missing school or work days (34).

### Other Youth Mental Health Services

The young mental health reform started in Australia has permeated to different zones of the world, including the UK, Ireland, Canada, USA, Europe, and Asia embracing unique, culturally sensitive models (70, 72). Some examples are given below and a systematic list of integrated services for young people (aged 10–30 years) along with their characteristics (year of setup, number of services, age range, targeted issues, position in care system, and number of young people accessing the service) is depicted in **Table 2**.

**Table 2:** Evaluation studies on mental health programs for young people (aged 10–30 years) that include a mental health function and are integrated—in that they bring together or provide a range of physical health, mental health, and social service foci. Adapted from Ref. (71).

Your mental health services	Country	Number of services	Established	Age range	Target issues	Position in care system	People accessing the service
Jigsaw	Ireland	10	2008	12–25	Mental health	Primary care	8,000
Headspace	Australia	110	2006	12–25	Mental and physical health	Primary and secondary care	80,000
Maisons des Adolescents	France	104	2004	11–25	Mental and physical health	Primary and secondary care	310,000
Youth One Stop Shops	New Zealand	11	1994	10–25	Mental and physical health	Primary care	34,000
Foundry	Canada	11	2015	12–24	Mental and physical health	Primary and secondary care	912
Youth One Stop Shops	Ireland	4	2009	11–25	Mental and physical health	Primary care	NA
ACCESS Open Minds	Canada	Underway					
Integrated Collaborative Care Team	Canada	Underway					
Your Choice	New Zealand	1	2008	10–24	Mental health	Primary care	976
Community Health Assessment Team	Singapore	1	2009	16–30	Mental health	Between primary and secondary care	601
The Well Centre	UK	1	2011	13–20	Mental and physical health	Primary care	934
Youthspace	UK	1	2011	16–25	Mental health	Unclear	NA
The Junction	UK	1	2003	11–18	Mental health	Secondary care	494
Supporting Positive Opportunities with Teens	US	1	2008	13–24	Mental and physical health	Primary care	1,729
Adolescent Health Service	Israel	NA	1993	12–18	Mental and physical health	Primary care	838
Rural Clinic for Young People	Australia	1	2010	12–18	Mental and physical health	Primary care	4,350
KYDS Youth Development Service	Australia	1	2005	12–18	Mental health	Unclear	1,600
Youth Stop	Australia	1	2010	12–25	Mental health	Unclear	20

#### Ireland

The reform of youth mental health in Ireland led to the Jigsaw care model in 10 communities (https://www.jigsaw.ie). This model was derived from Headspace and similarly focuses on young people aged 12–25. Initial evidence has shown that it is an accessible and effective mental health service in the community.

#### UK

In the UK, the creation of the “Youthspace” in Birmingham, a youth-based mental health service (http://www.youthspace.me), resulted in the commissioning of an integrated care pathway: Forward Thinking Birmingham (https://www.forwardthinkingbirmingham.org.uk). This children and young people mental health partnership offers integrated working, prioritizing both individual choice and access through drop-in clinics. Forward Thinking Birmingham is different from other models in that it targets those in the age group of 0–25. Furthermore, it is also focused on promulgating good mental health, resilience, and emotional well-being through the provision of information, training, and consultation. This will be achieved through the voluntary community sector, family support, and providing information in a wide range of media in order to reach the population of Birmingham. However, no published evidence exists as of now on the impact of this model of care.

Other approaches in the UK have attempted to ameliorate the quality of mental health services for young people in primary care or in CAMHS.

The Well Center Model (www.thewellcentre.org) is a multi-disciplinary model for young workers, counsellors, and general practitioners. In order to provide holistic care that is family oriented, evidence-based, and culturally sensitive, primary care requires an incorporated, integrated and collaborative approach between general practitioner surgeries, secondary care, schools, third-sector organizations, justice systems, and social services.

The THRIVE model (http://www.implementingthrive.org/about-us/the-thrive-framework/) was created by a joint effort of the Anna Freud National Centre for Children and Families and the Tavistock and Portman NHS Foundation Trust. This model is an integrated, personalized, and need-driven approach to providing children, young people, and their families with mental health services. The focus is set on the prevention of mental disorders and the promotion of psychological well-being. Through a system of shared decision-making, children, young people, and their families can be empowered *via* active involvement in decisions about their care (73). Initial evidence proposes that the THRIVE approach can improve the mental health of children and young people.

#### Canada

Canada has joined the global youth mental health service movement with consolidated efforts from the Mental Health Commission of Canada, including various regional services interventions (e.g., YouthCan Impact in Ontario; Foundry in British Columbia). The special investment was recently made in the fields of service transformation research and evaluation, as shown in the ACCESS project for persons aged 12–25 (www.accessopenminds.ca) (74). Interestingly, the ACCESS project supports the view that any single model of service transformation for children and young people is not implementable over the geographic, political, and cultural diversity of this nation. Hence, the best way to overcome such obstacles is to steer test variations of a model of transformation customized to contextual scenarios before scaling it up or implementing a type of service that has been developed and imported from another country (74). The ACCESS approach encompasses different domains: promotion, prevention, intervention, and research and evaluation. ACCESS differs from Headspace since it doesn’t propose the creation of a new system of care for young people. Rather, it proposes the radical creation of a transformed youth mental healthcare system that is embedded in the existing care system. The fundamental standards of this transformation should be introduced on reducing to the lacunae that are impeding access to timely and adequate care for young people (12–25 years of age) who are presenting with the whole range of mental health problems, as discussed above (74).

### Outcomes

In a recent systematic review, 43 evaluation reports examine at least one aspect of the outcome of interest for integrated mental health services for children and young people:

- Access: most integrated services report attracting youngsters in the mid-older adolescent age range and traditionally underserved populations, including minorities. Levels of distress of young people accessing the services are defined and described variably across these evaluation reports. Presenting problems are commonly identified with mental health and psychosocial difficulties and less likely with physical health, educational, and vocational issues. Individual counselling is the most commonly described intervention following access to these services (75).
- Symptomatic and functional outcomes; clinical outcomes are reported for 7 out of 43 reports only (75) and mostly in pre–post study designs. In the Your Choice service study (**Table 2**), young people experienced critical decreases in symptoms and substance use as well as amelioration in functioning (75). In the Youth One Stop Shop service (**Table 2**), 58% of young people who presented with some difficulties experienced improvements in the short term. According to an evaluation of the Jigsaw service (**Table 2**), 62% of 17- to 25-year-olds displayed an improvement in their level of well-being and functioning. A study by Youthspace (**Table 2**) found that 58% of young people experienced an improvement in mental health and well-being. Comparative studies, such as the most recent evaluation of Headspace, found some promising results. For instance, over 20% of young people encountered a clinically significant or reliable decrease in trouble that was greater than a compared external group of young people who had not received any treatment (75). However, the effect size was observed to be quite small (*d* = −0.11) (75). The results are overwhelmingly positive when a survey design is used in the evaluation.
- Satisfaction, acceptability, and appropriateness (75). Whenever estimated, elevated levels of service users’ satisfaction are commonly revealed. A common finding is that young people find (and value) that these services are accessible, acceptable, and appropriate:
  - Having a convenient location (access to easy transport was noted as being valuable);
  - Being youth-friendly (staff and environment) and welcoming;
  - Being staffed by youngsters;
  - Having timely appointments;
  - Being affordable;
  - Maintaining confidentiality and privacy;
  - Having many incorporated services accessible in one spot, with non-mental-health-related signage;
  - Delivering sheltered and appropriate interventions inside a positive and resilient- based framework (72).

To summarize, the evidence for mental health services for people aged 0 to 25 indicates that:

- High-order (WHO) standards overseeing the development of youth-friendly health services are available;
- The building blocks for reforming youth mental services began with the early intervention for psychosis in adolescents and young adults;
- The UK has unparalleled central resources for early detection and treatment of individuals aged 14–35 who are experiencing emerging serious mental disorders;
- Early interventions in bipolar, depressive, and other mental disorders may be feasible;
- The youth mental health reform started in Australia has penetrated to different territories of the world, including the UK, Ireland, Canada, USA, Europe, and Asia;
- There are different models of care spanning the establishment of a new system of care (Headspace) or the transformation of the care system (ACCESS);
- One-stop youth-friendly mental health services can improve access, symptomatic, and functional outcomes and satisfaction of the service users;
- The integration of physical and mental health in youths can have synergic benefits;
- Integrated mental health services mostly focused on adolescents and young adults (12–25).

## Challenges

Although there has been converging evidence that children and young people need integrated mental health services during the developmental period, there are still some challenges. First, in spite of significant efforts to develop holistic services and programs for youth-to-adult transitions, and also following nearly two decades of youth mental health research, there remains an absence of standards and models of care guiding research, service planning, and delivery for children and adolescents progressing from CAMHS to AMHS (47). No single example or model that can be considered to establish the best practice is provided (72). Second, the evidence of the effectiveness of integrated mental health models of care for children and young people remains modest. The types of evaluations described in the Outcome section vary in quality, but they are overall classified as Level IV evidence only, according to National Health and Medical Council levels of evidence (75): “evidence obtained from case series, either post-test or pre-test and post-test.” No high-quality pragmatic randomized controlled trial has yet been published in the international scientific databases (76), not even for the most established models of care. However, some trials are underway, which demonstrates that it is feasible to run these types of studies in this field (75). Third, cost-effectiveness studies are similarly lacking. This may be particularly concerning given the fact that the reference model, Headspace, required substantive financial funding by the Australian government in order to establish brand new youth mental health services across the country. Besides, 40% of Headspace patients are excessively complicated or too unwell to profit by the program. Thusly, more specialized and intensive healthcare components should now be financed, gathered, and integrated horizontally with Headspace and other important pieces of the health and social system vertically (34). This would further increase the costs for upkeeping Headspace-like models of care. Until recently, there has been very little cross-national focus on how mental health services for children and youth are organized and financed (77). In the current financial climate and growing demand for mental health services among young individuals, it is important to understand international best practices that can improve service accessibility and reduce financial and organizational barriers to availing services at the patient level (77). In this scenario, the Canadian approach (ACCESS) focusing on transforming mental health, as opposed to creating brand new services, may be more feasible. This could be further facilitated by the existing national early detection and intervention services for psychosis within the UK. Notably, this platform is already demonstrating scalable impact for taking care (across CAMHS and AMHS) of both children and young adults aged 14 to 35. Fourth, an extra challenge is that suitable clinical and treatment response to the earliest signs of disorders in young people is yet to be completely clear. This lack of knowledge is problematic because the risk-to-benefit ratio of specialist early care is totally different in the wider subclinical, primary and secondary care population from that in the youth mental health services wherein these interventions have been developed. Treatment challenges have also been observed for the most established early intervention field for psychotic disorders (78). Fifth, the challenges mentioned above are even more pronounced for people below the age of 12, including those of perinatal, infancy, and early childhood age. In fact, the existing evidence for developing integrated mental health services for CAMHS and AMHS nearly focuses entirely on people between 12 and 25 years of age, with very few special exceptions that still require demonstration of feasibility and impact.

To summarize, the main challenges for mental health services for people aged 0–25 are:

- There are no standards and no single example can be considered to constitute best practice;
- The evidence of the effectiveness on mental health outcomes is modest; there are no RCTs;
- Cost-effectiveness studies are similarly lacking;
- Appropriate clinical and treatment response yet to be entirely clear;
- Very little evidence for individuals aged 0–12.

## Conclusion

The focus of many emerging international health agendas is on the mental health of young people (2). An important strategy to enhance global health outcomes is to invest in identifying and addressing the mental health needs of vulnerable children and young people (79). There is a growing consensus that children and young people need youth-friendly mental health services that are sensitive to their unique stage of clinical, neurobiological, and psychosocial development. Evidence has confirmed that the transitional phase from adolescence into young adulthood (12–25) represents a core window of opportunity for improving the outcomes of mental disorders. Conversely, there is only limited evidence that detection and intervention in the lower age (0–12) range is feasible and effective. The current configuration of mental health services split between CAMHS and AMHS is highly inefficient since it does not reflect state-of-the-art scientific evidence and produces barriers to access and treatment, and poor retention rates that impede early intervention approaches for those in need.

While different possible youth-friendly mental health models can be considered, there is a growing consensus that the focus should be kept on early detection and intervention models within the community that target both adolescent and young adults. The most successful early intervention paradigm that fully integrates adolescents and adult mental health services alike is the prevention and early treatment of psychosis. Over the past decade, the UK has implemented nationwide first-in-class early intervention services for psychosis. Therefore, it may be possible to leverage these UK early intervention templates in order to refine the next generation of youth-friendly mental health services that target the needs of adolescents and young people experiencing early stages of other mental disorders (e.g., depression, bipolar).

## Author Contributions

The author designed the study, obtained financial support, and conducted the literature review, data extraction, and the interpretation of the findings.

## Funding

The study was funded by the Healthy London Partnership (https://www.healthylondon.org/). The funder had no influence on the design of the study or interpretation of the results.

## Conflict of Interest Statement

The author declares that the research was conducted in the absence of any commercial or financial relationships that could be construed as a potential conflict of interest.
